# Characterization of Wood and Graphene Nanoplatelets (GNPs) Reinforced Polymer Composites

**DOI:** 10.3390/ma13092089

**Published:** 2020-05-01

**Authors:** Zainab Al-Maqdasi, Guan Gong, Birgitha Nyström, Nazanin Emami, Roberts Joffe

**Affiliations:** 1 Department of Engineering Materials and Mathematics, Luleå University of Technology, SE-971 87 Luleå, Sweden; nazanin.emami@ltu.se; 2 Rise Sicomp AB, Fibervägen 2, SE-941 26 Öjebyn, Sweden; guan.gong@ri.se; 3 Podcomp AB, Skylvägen 1, SE-943 33 Öjebyn, Sweden; birgitha.nystrom@podcomp.se

**Keywords:** graphene nanoplatelets (GNPs), nanocomposites masterbatch, wood polymer composites (WPC), energy transport, high density polyethylene (HDPE)

## Abstract

This paper investigates the utilization of commercial masterbatches of graphene nanoplatelets to improve the properties of neat polymer and wood fiber composites manufactured by conventional processing methods. The effect of aspect ratio of the graphene platelets (represented by the different number of layers in the nanoplatelet) on the properties of high-density polyethylene (HDPE) is discussed. The composites were characterized for their mechanical properties (tensile, flexural, impact) and physical characteristics (morphology, crystallization, and thermal stability). The effect of the addition of nanoplatelets on the thermal conductivity and diffusivity of the reinforced polymer with different contents of reinforcement was also investigated. In general, the mechanical performance of the polymer was enhanced at the presence of either of the reinforcements (graphene or wood fiber). The improvement in mechanical properties of the nanocomposite was notable considering that no compatibilizer was used in the manufacturing. The use of a masterbatch can promote utilization of nano-modified polymer composites on an industrial scale without modification of the currently employed processing methods and facilities.

## 1. Introduction

The growing awareness of the environment and the demands for increasing the sustainability in resources and industries have urged the development of bio-based materials for use beyond their structural applications. Production of bio-based materials such as wood polymer composites (WPCs) with added functionalities (e.g., thermal or electrical conductivities) can partially answer these demands. This can be made possible by profiting from the advances in the research and technology of nanomaterials.

Modification with graphene and graphene derivatives has been realized recently as a way to increase the mechanical properties of polymers as well as wood polymer composites and/or to impart functionality, such as thermal/electrical conductivity or electromagnetic shielding interference [1,2,3]. The application of graphene nanoplatelet (GNPs) in composites is foreseen to grow between 2018 and 2025 by about 40% as it is being increasingly researched for composite-utilizing industries, such as construction, aerospace, and automotive sectors [4,5]. Available studies have shown the potential of modifying wood composites with nano-reinforcement to improve properties making them candidates for advanced construction applications [6,7].

Incorporation of different graphene-based derivatives has shown positive effects on the thermal, electrical, and mechanical properties for a variety of thermoplastics, such as polylactic acid (PLA) [8], polyvinyl alcohol (PVA) [9], polyethylene terephthalate (PET) [10,11], polyamide (PA) [12], and others. For example, the mechanical properties of high-density polyethylene (HDPE) were continuously enhanced with the addition of GNPs up to 15 wt% [13]. Moreover, 16% improvement in thermal conductivity of HDPE nanocomposites prepared by a melt-mixing process was achieved at a graphene content of only 1 wt% [14]. Similarly, GNPs were found to improve the properties of wood polymer composites (WPCs) [15,16]. The addition of 12 wt% of GNP resulted in more than a 30% improvement in flexural properties of the WPC and above a 200% increase of the thermal conductivity compared to the control WPC sample [15]. However, in the case of wood polymer composites, a compatibilizer is usually employed to increase the adhesion between the wood and the polymer. Compatibility of the nanofillers with the polymer matrix has also been reported to be increased by the addition of a compatibilizer [17,18].

One of the main challenges of reinforcing polymers with graphene is its tendency to aggregate and restack which results in a reduced graphene/polymer contact area and limited load transfer efficiency. It also reduces their efficiency to the added functionality of the composites and, thus, hinders their utilization in the various applications [6]. Selection of an appropriate production method of the nanocomposite [18] and chemical functionalization of graphene [8] can mitigate the abovementioned problem. Solution mixing and in situ polymerization of the polymers in the presence of GNP are found to be efficient processes to produce well-dispersed nanoparticles in the polymers [19]. Nonetheless, among other disadvantages, these processes are not easily scaled up and difficult to provide the large amounts of nanocomposites required for industrial-scale production. On the other hand, melt mixing is a more cost-effective method that utilizes conventional processing techniques of polymers, such as screw extrusion. The shear forces applied by the rotating screws contribute to the separation of the graphene sheets in the platelets to achieve the dispersion of the nano-reinforcement. This technique, though, would still require dealing with the dry form of the GNP, which can be hazardous and also challenging to feed into the equipment due to its low bulk density [20].

Recently, tackling these problems have been made easier with the emerging technology of the patented nanocomposite masterbatches [21]. Such materials are polymers with high content of the nanoparticles (graphene, carbon nanotubes, or clay platelets) which are well dispersed using scalable, environmentally friendly techniques [22] and ready to be mixed with base polymer to prepare the composite with desired nano-reinforcement content by melt mixing. This technique ensures easy, improved dispersion due to the additional step of shearing the compound and high throughput of materials while at the same time achieving improved mechanical and functional properties. Despite the existence of studies in which the masterbatch technique is used (at lab or pilot scales) in the preparation of the polymer nanocomposites, there is no study (to the best of authors knowledge) that reports its use in developing GNP modified wood polymer composites using melt mixing with limited amounts of coupling agent.

In this work, a commercial masterbatch of GNPs in HDPE was used to enhance the physical-mechanical properties of the polymer and wood flour-reinforced polymer composite. The graphene platelets in the masterbatch are functionalized at the edges for improved compatibility with the polymer while preserving the characteristics of graphene in the bulk. Polymer nanocomposites were manufactured without the addition of a coupling agent and in a single run through the extruder while a limited amount of the coupling agent was used in the preparation of the WPC. The synergistic effect of reinforcing the polymer with varying amounts of wood and GNPs was also demonstrated. The choice of material and the production technique contribute to the development of sustainable industry and products ready for upscaling without the need to develop new, more sophisticated, and expensive processes.

## 2. Materials and Methods

### 2.1. Materials

Thermoplastic of high-density polyethylene (MG9647S) in the form of pellets was purchased from BOREALIS AG (Vienna, Austria). Two HDPE-based masterbatches with edgily functionalized carbon platelet additives were used. The first masterbatch is heXo HDPE1-V20/35 (further denoted as M1, from NanoXPLOre, Montreal, QC, Canada) with 35 wt% dispersed graphene nanoplatelets having average thickness of 20 nm, flake size of 50 μm, and the number of layers in one platelet is ~40. The second masterbatch contains 25 wt% Graphene Black^TM^ 3X (further referred to as M2, from NanoXPLOre, Montreal, QC, Canada) having 6–10 layers in the platelet with a flake size of 38 µm. According to the manufacturer, the functionalization of the platelets resulted in an oxygen content that did not exceed 7 wt% in both masterbatches. The wood flour (WF, from Scandinavian Wood Fiber AB) is sawdust of spruce and pine wood with 75% of its particles in the size range 200–400 µm. The term “wood fiber” is also used in the following sections to refer to the particles of the wood flour due to the relatively large aspect ratio of these particles. More than 60% of the particles have an aspect ratio larger than 3 (see Figure S1 in Supporting Information). Maleic anhydride-grafted high-density polyethylene (MAPE), E265, provided by DuPont (DuPont, Wilmington, DE, USA), was used as the compatibilizer in WPC.

### 2.2. Fabrication of the Composites

Nanocomposites with selected amounts of GNPs were manufactured by feeding the masterbatch with neat polymer into a co-rotating twin screw extruder ZSK25 (Krupp Werner and Pfleiderer GmbH (now Coperion), Stuttgart, Germany) with an l/d of 44 (a schematic of the extruder depicting the different zones is shown in Figure S2). The operation parameters and temperatures of the extruder are presented in Table 1, while the sample nomenclature with its composition is presented in Table 2. These parameters were used to manufacture all of the composites (reference WPC, as well as GNP-modified), the nano-modified WPC were prepared using masterbatch M1. Prior to manufacturing, wood flour was dried for 8 h in an oven at 100 °C then fed through the side feeder into the extruder while all other components (HDPE, MAPE, and masterbatch) were mixed and fed through the main feeder. The WPC (with and without GNPs) were run through the extruder twice while the nanocomposites went a single run through the extruder, as shown in Table 1.

Measured amounts of the extrudate were heated in an infrared oven for 30 min at 220 °C, then plates were pressed using a conventional 310-ton compression molding press (Fjellman Press AB, Marinstad, Sweden). The resulting circular plates having the dimensions of 320 mm in diameter and 4 mm in thickness were then cut into rectangular samples using a waterjet followed by drying in the oven at 80 °C for 8 h. For the neat polymer, pellets were directly melted, and compression molded under the same conditions except that they were not extruded.

### 2.3. Characterization

#### 2.3.1. Morphology

The composites were characterized by using JEOL JCM-6000 Neoscope scanning electron microscopy (JEOL Technics LTD, Tokyo, Japan) on test-fractured surfaces or freeze-fractured surfaces sputter-coated with a thin layer (<15 nm) of conductive element (gold or palladium).

#### 2.3.2. Tensile Test

Rectangular samples with dimensions of 15 mm × 200 mm (100 mm gauge length) were tested on an Instron 3366 universal testing machine (Instron^®^, Norwood, MA, USA) equipped with 10 kN loadcell and pneumatic grips. Tests were performed in an extension control mode with a cross head speed of 5 mm/min for the PE0/nanocomposites and 2 mm/min for the composites with WF (this corresponds to strain rates of 5%/min and 2%/min, respectively). Strain was measured by a standard Instron extensometer (Instron 2620-601, Instron^®^, Norwood, MA, USA) with a base length of 50 mm. An initial loading-unloading step with a maximum applied strain up to 0.25% was performed to measure initial stiffness of undamaged material. Assuming the linearity of behavior in this region, stiffness was obtained from the slope of the unloading segment of the ramp within a 0.20–0.05% strain interval. Subsequently, the sample was loaded with the same rate until limitation of the extensometer was reached and test was finished (this limit did not necessarily break the samples). The yield stress was obtained by the intersection point of the stress–strain curve with the 0.2% offset strain line following guidelines of the ASTM D638-14 [23]. A minimum of five valid tests were conducted and the presented results are the average of all recorded values.

#### 2.3.3. Impact Test

Impact strength was determined by performing impact test on Charpy-type setup (WPM Werkstoffprüfsysteme Leipzig GmbH, Markkleeberg, Germany). This apparatus is equipped with a hammer capable of providing a maximum breaking energy of 14.7 J for a sample resting on supports separated apart by 30 mm with an impact velocity of 3.8 m/s. The equipment was calibrated according to the ASTM standard D6110-10 [24] and the energy value readings were corrected to the windage of the hammer and the energy losses due to friction. Specimens (five per test) of dimensions of 125 mm × 12.7 mm (length × width) were notched to a V shape notch (angle of 45°, radius of 0.25 mm, depth of ~2.5 mm) manually using a razor blade to the possible accuracy on one edge and impacted on the opposite unnotched edge. The accuracy of the notch was not taken as assessment criteria for the validity of the specimen for test. The reported impact strength is the result of dividing the net breaking energy by the total area of the developed crack (cross-section area of the unnotched portion of the specimen).

#### 2.3.4. Flexural Test

Following the guidelines of ASTM D790-17 [25], flexural properties were determined by means of a three-point bending test setup on an Instron 4411 machine (Instron^®^, Norwood, MA., USA) equipped with 5 kN loadcell. Five specimens having dimensions 80 mm, 16 mm, and 4 mm (length, width, and thickness) with a support span distance-to-thickness ratio of 16:1 (64 mm) were tested with a cross-head speed assuring straining the outer fibers by a rate similar to that of the tensile test (5%/min and 2%/min for the nanocomposites and WPCs, respectively). Conditioned samples were also tested to study the effect of moisture on the flexural properties.

Conditioning was performed on three additional specimens of each batch of material. Prior to the test, samples were dried in the oven (Termaks AS, Bergen, Norway) at 40 °C until the difference between two successive measurements was negligible (<0.2%) then edge-sealed with water repellent commercial Silyl-modified polyether (Casco from Sika Sverige AB, Spånga, Sweden) to ensure one-dimensional moisture uptake. Samples were weighed and placed in a plastic container with water at 40 °C (specimens were not immersed but suspended over the water with a measured relative humidity RH = 99%). Moisture uptake was monitored by frequent measurements of the sample weight on an AG245 analytical balance (from Mettler Toledo, Columbus, OH, USA) and water content was assessed by the relative difference in mass before and after the exposure. It is worth noting that during weighing or testing, the relative humidity in the lab was different than the conditioning environment which might have caused uncontrolled diffusion of moisture into or from the specimen. However, care was taken to minimize the exposure of samples to these conditions. The moisture content of the samples at the time of test are found in Table 3 below together with time required for saturation.

#### 2.3.5. Crystallinity and Thermal Properties

DSC 821e differential scanning calorimeter (Mettler Toledo, Columbus, OH, USA) was used to determine the degree of crystallinity as well as the characteristic temperatures of the polymer. Samples of weight 5–12 mg, encapsulated in aluminum pans with pierced lids, were subjected to a thermal profile in nitrogen atmosphere (gas flow of 80 mL/min). Temperature was raised from 25 °C to 200 °C (past the theoretical melting point of HDPE) in a rate of 10 °C/min and kept under isothermal conditions (200 °C) for 5 min to erase thermal history, then cooled to room temperature (25 °C) with a rate of 20 °C/min using liquid nitrogen. A second heating run was performed with a heating rate of 10 °C/min from which the thermal properties where determined. Melting onset and peak value along with crystallization temperatures were determined from the endothermic and exothermic peaks of the DSC curve, respectively. Crystallinity was calculated from the heat of fusion obtained by integrating the endothermic peak between 60 °C and 180 °C according to the following Equation (1):

(1) $$\%X_{c}=\frac{\Delta H_{f}}{\Delta H_{f}^{0}}\times \frac{1}{1-W_{f}}\times 100,$$

where $\Delta H_{f}$ is the measured melting enthalpy of sample, $\Delta H_{f}^{0}$ is the theoretical melting enthalpy of 100% crystalline PE (293 J/g obtained from [26]), and $W_{f}$ is the weight fraction of the reinforcement.

Hot disc thermal constants analyzer TPS 500 (Hot Disc^®^, Gothenburg, Sweden) was used to determine the thermal conductivity and thermal diffusivity of the material. Squared 40 mm × 40 mm samples with a nominal thickness of 4 mm were tested at room temperature using the room temperature sample holder. Based on the available probing depth, the sensor 5465 (enclosed in Kapton insulation films, Hot Disc^®^, Gothenburg, Sweden) with the radius of 3.2 mm was selected. The sensor was placed between two pieces of sample material with proper pressure applied to ensure full contact between the sensor and the surfaces of the sample. Measurement time was 2.5 s with a heating power of 96 mW assuming isotropic material. The experiment was performed in a series of 5 measurements per specimen with a waiting time of 15 min between the measurements to allow the sample to reach equilibrium. Values presented are the average of these five measurements. It is worth noting, though, that the results shown here are valuable for showing the general trends rather than absolute values and more accurate results could be achieved if the sample-to-sensor size was better optimized.

## 3. Results and Discussion

### 3.1. Morphology

**Figure 1:**
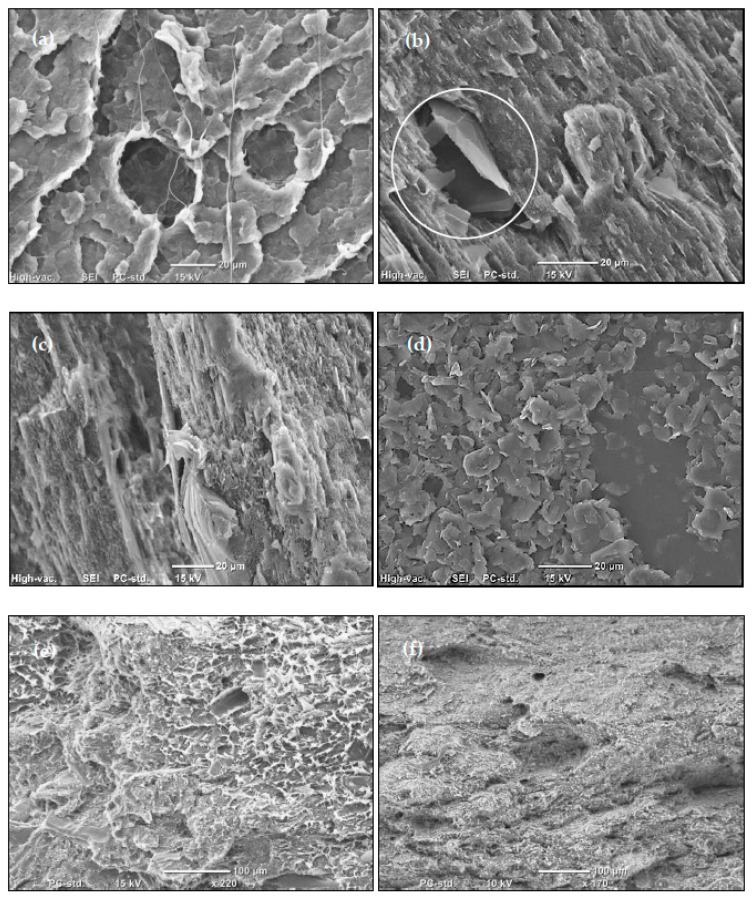
SEM of fracture surfaces of samples showing the morphology change with the addition of GNP: (**a**) PE0; (**b**) PE15-M1; (**c**) a close-up on a PE6-M1 of a large platelet showing the layered structure; (**d**) a cluster of agglomerated particles in PE15-M1; (**e**) fracture surface of 40WPC; and (**f**) fracture surface of 40WPC15. (**a**–**d**) freeze-fractured; (**e**,**f**) test-fractured (as-received, flexural test).

Freeze-fractured surfaces (samples broken after immersion in liquid nitrogen) of the samples under SEM are shown in Figure 1. The morphology of the sample is changing from a topological surface, with fibril-forming fracture for neat polymer (Figure 1a), to a more smoothened surface and brittle-like fracture for the GNP-reinforced materials (Figure 1b). From the Figure 1, it could also be seen that the particles are well distributed in the polymer as they span the image evenly. The large folded particle is also seen in the image (enclosed in the white circle) which might act as a defect area for a premature failure due to the weak bonding with the polymer and the void surrounding it can act as a water pocket. However, despite the generally good distribution, inhomogeneity observed in Figure 1c implies that the dispersion state can still be improved. The image shows a particle with thickness about the size of the scale bar which is much higher than the thickness provided by the supplier, indicating that this particle is a restack of multiple platelets. This would also mean that there is a greater potential and room for improvement if better dispersion can be achieved. Moreover, Figure 1d shows a cluster of platelets agglomerated in one place at 15 wt% GNPs loading. This suggests the greater challenge to disperse the particles at high loadings of nano-reinforcement.

Due to the large difference in the size scale between the two types of reinforcements (wood and GNPs) it is difficult to examine the distribution of one without losing information about the other in the image. However, the presence of the nanoparticles is obvious even at lower magnification by their effect on the morphology of the fracture surface of the wood samples. The plastic deformation of the matrix (appearing as white protrusions) in the 40WPC are replaced by a smoother surface in the 40WPC15 sample (Figure 1e,f, respectively).

In general, it can be stated that the bonding between the matrix and the platelets is good despite the absence of compatibilizer in the nanocomposites, and the GNPs seem to be well embedded in the polymer. Occasional occurrences of voids, and the presence of the agglomerates, might cause premature failure of specimens at random locations especially if they were close to the edges.

### 3.2. Tensile Properties

**Figure 2:**
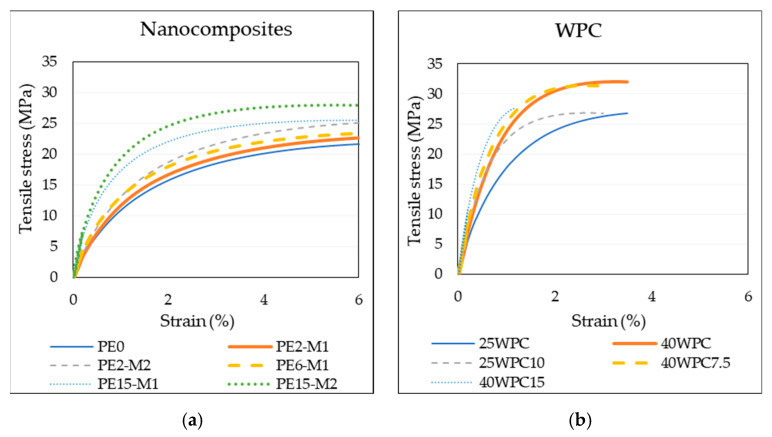
Tensile stress–strain curves of (**a**) nanocomposites, and (**b**) wood polymer composites. Colored version of the graphs can be found online.

Representative stress–strain curves of the polymer and composites are plotted in Figure 2. Regardless of the obvious change of response towards stiffer and stronger materials for the nanocomposite compared to the neat polymer, samples did not break during the test even after exceeding 8% strains. For the sake of fair comparison, the curves are presented until 6% for all the samples. There is a gradual increase in stiffness with the increase in GNPs content (numerical values are tabulated in Table 4). It is also noticeable that masterbatch M2 is more effective in reinforcing the polymer than M1 that have higher number of graphene layers in the platelet. Improvement by addition of 2 wt% GNP of masterbatch M2 in the polymer competes, or even exceeds, that of material with 6 wt% GNP of the M1 in some cases. For the same content of GNPs in polymer there is always higher improvement of properties for the M2 system over that of M1. This is because M2 platelets have fewer graphene layers and, thus, the aspect ratio of the platelets and graphene-polymer contact surface area are larger than those for M1. With properties of neat polymer being the reference value, a maximum improvement of tensile stiffness and yield stress (140% and 79% respectively) was achieved for sample PE15-M2 while the lowest increase (12% and 2%, respectively) was obtained for PE2-M1.

In an earlier study [27], the increase of stiffness of HDPE reinforced with approximately similar amounts of GNPs as used in this study was inferior to the values reported here. Considering the lower properties of the starting materials in [27], and the use of large amounts of compatibilizer one could expect more significant improvement upon the addition of the reinforcement. Furthermore, avoiding working with the nanoparticles in their hazardous dry form favors the use of the masterbatch despite the similarity of the used processing method.

Similarly, WPC witnessed improved tensile properties with the addition of the GNPs. These are presented in the stress–strain curves (Figure 2b) as well as in Table 4. In this case, though, the composite became more brittle and the elongation at break was reduced (between 1% and 4% for 40WPC15). Regarding the WPC, it is possible to separate the effect of WF from that of the GNPs. The improvement for the 40WPC15 material for the stiffness and yield stress is 219% and 146%, respectively, if compared to PE0, while improvements of the same properties were 50% and 14% with respect to 40WPC.

Earlier research [7,13] reported a decrease in the properties of the WPC after a certain additive content (typically below 5 wt%) and attributed that to the possible agglomeration of these particles that act negatively on the mechanical performance of the composite. However, it does not seem to be the case here and the improvement is confirmed until up to 15 wt% content of nano-reinforcement.

These results are in accordance with the microstructure studied via SEM where good distribution of the particles was evident and their compatibility with the polymer was satisfactory despite the presence of some defects. The small scatter of the values confirms that not only is there an even distribution of the particles but also an even distribution of the present defects. It also indicates that a larger improvement in the mechanical properties could be achieved should the dispersion state be further enhanced.

### 3.3. Impact Strength

**Figure 3:**
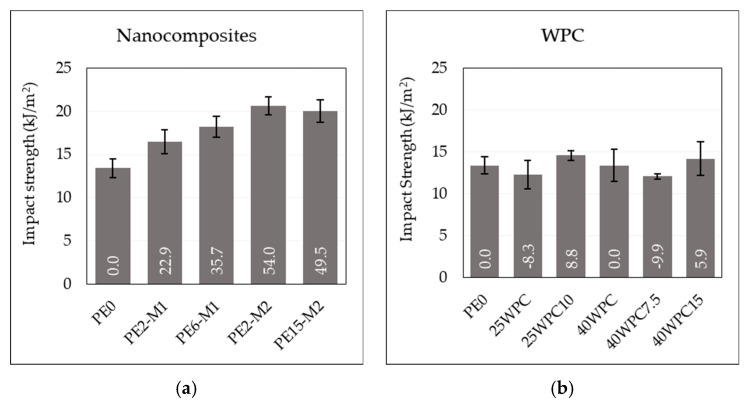
Impact strength of (**a**) the nanocomposites, and (**b**) the wood polymer composite at different graphene and wood fiber loadings. Data labels represent the percentage change with respect to neat polymer.

**Figure 4:**
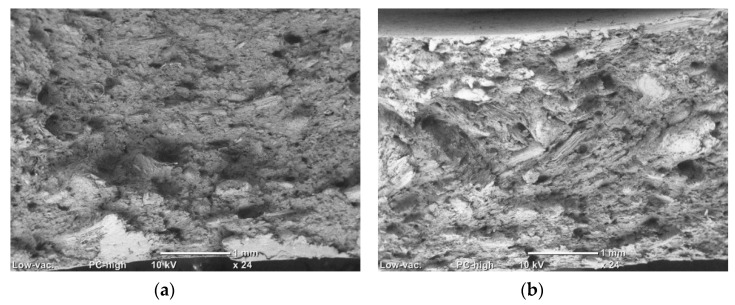
SEM images of the impact fracture surface of: (**a**) 40 WPC; and (**b**) 40WPC7.5.

The impact strength of the nanocomposites and the WPCs are shown in Figure 3. The addition of the GNPs has a positive effect on the impact strength of the polymer. A maximum improvement of around 54% was noticed upon the addition of 2 wt% of GNP of the thinner particles (M2) which exceeds that of 6 wt% of M1 (for which improvement was > 35%). For the same GNP content, improvement in the impact strength of the M2 masterbatch was more than twice that of M1, which indicates a larger surface area for contact that requires higher energy to separate. The rigid particles may induce new forms of energy dissipation that leads to delayed fracture. These can be in the form of pull-outs, crack pinning [28], or bridging the polymer chains and redirecting the crack to new paths before the final fracture. Layer sliding is another form of toughening associated with the layered reinforcement [29], due to the consumed energy in overcoming the forces binding the layers in the platelet. Such an increase in the impact strength suggests the relatively good dispersion of the GNP additives (see Figure 1 for SEM micrographs) which has been reported to affect this property [11] in contrast to the usual decrease accompanying a particulate composite resulted by the loss of polymer toughness [30]. However, at higher loadings, where agglomerations are expected, the pull-out of large agglomerates is easier and requires less energy and, thus, the resistance to crack propagation is reduced [31]. It is also possible that these agglomerates act as new sites for crack initiation distributed over the volume of the specimen that enhance energy dissipation and mitigate the localized crack propagation and, thus, competing mechanisms of toughening occur. The addition of wood flour results in marginal or negative effects on the impact strength of the polymer, which is only compensated at high GNP loadings. Figure 4 shows fracture surfaces of two WPC samples with and without GNP reinforcement. Despite the clear difference in the surface features upon the addition of the GNPs, where the surface looks more disturbed at the presence of the GNPs (indicating higher energy needed to create it), these two samples showed contradicting values of impact strength. This explains the competing mechanisms mentioned above.

### 3.4. Effect of Moisture on Flexural Properties

**Figure 5:**
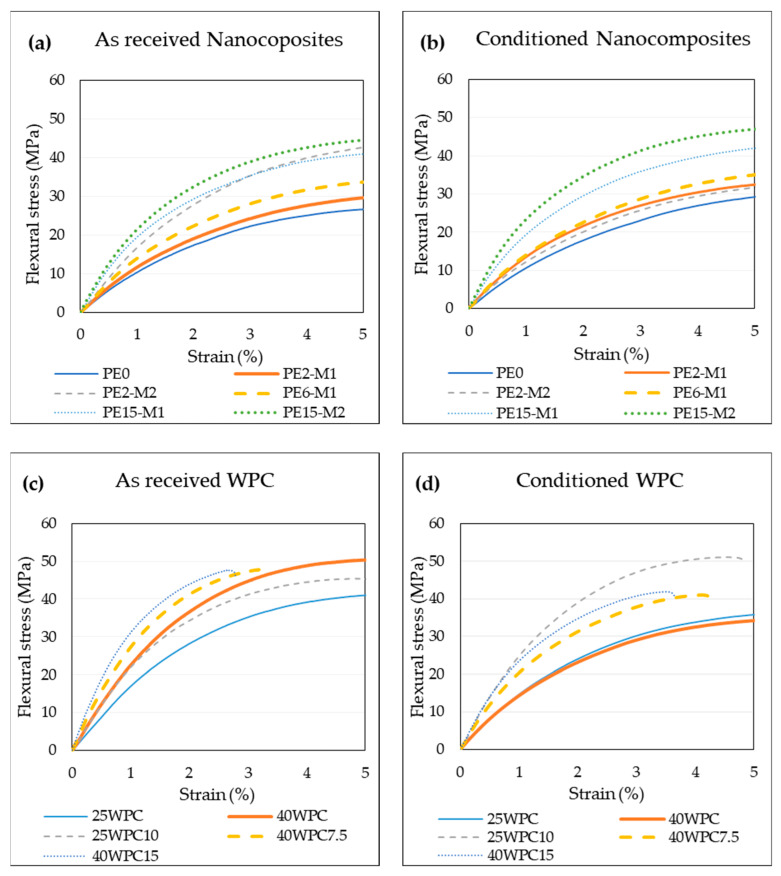
Flexural stress–strain curves of the as received and conditioned materials (at T = 40 °C and RH = 99%). Colored curves can be found in the online version of the article.

**Figure 6:**
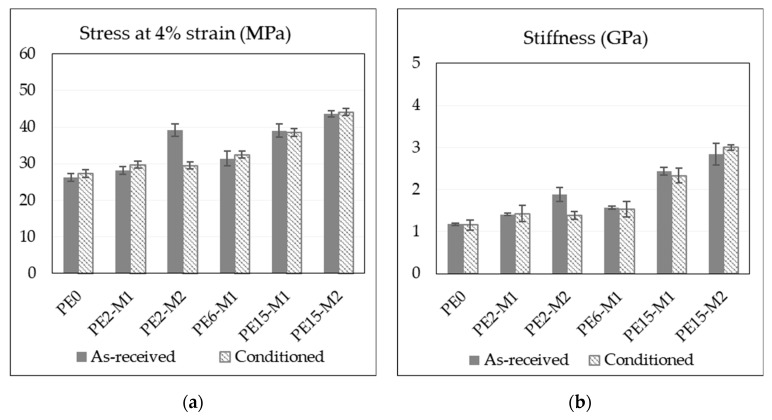

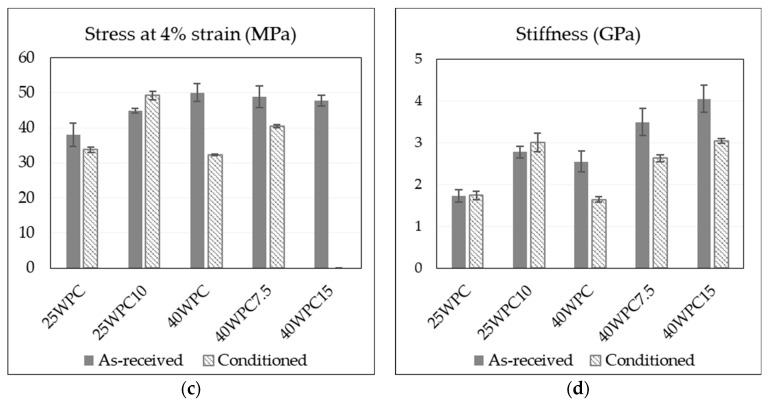
Effect of moisture on the flexural properties of the materials: (**a**,**b**) flexural stress and stiffness for the nanocomposites; (**c**,**d**) flexural stress and stiffness of WPCs. Sample 40WPC15 broke at average strains below 4% (3.47%) registering an average maximum stress of 41.7 MPa.

**Figure 7:**
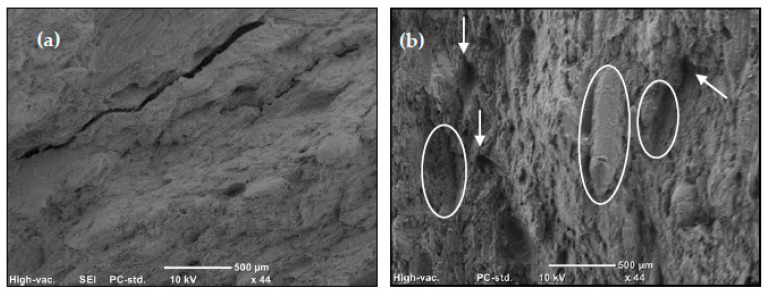
Flexural fracture surface before and after moisture uptake. (**a**) as-received 40WPC15; (**b**) conditioned 40WPC (at T = 40 °C and RH = 99%). See significance of the marks in the text.

It is well known that elevated moisture content in the polymer might influence its mechanical performance. Water acts as a plasticizing agent that decreases the mechanical properties [32] (e.g., stiffness and yield stress). This effect was investigated by measuring the flexural properties of materials before and after exposure to moisture. Figure 5 shows representative flexural stress–strain curves of the nanocomposite and the WPC, before and after moisture uptake. In general, trends similar to those from the tensile tests are observed here. The materials become stiffer with the increased content of GNPs and the masterbatch of fewer layers of graphene in the platelets (M2) outperforms the other. For the WPCs, the increase in the content of any of the reinforcements leads to an improvement in the composite performance. The effect of moisture on the properties of the nanocomposites is not so significant. Except for sample PE2-M2, where the curve is lower, indicating degraded properties, the general trend seems to be the same and effect of moisture on the materials can be regarded as marginal. Despite the relatively large difference between the moisture content of the nanocomposites (ranging from below 0.05% for PE2-M1 to more than 0.6% for PE15-M2), the results do not seem to be affected. This is expected since none of the nanocomposite constituents tend to absorb water and the effect is limited to the possible penetration of water molecules into the voids at high GNP content. On the other hand, the moisture effect on WPCs is more pronounced. The moisture-saturated wood in the composite decreases the flexural properties and the presence of the nanoplatelets seems to restrict this effect. Since only three conditioned samples were tested, scatter was larger in these samples and the stress–strain curves in Figure 5 might not be fully representative and do not show the scatter, it is therefore more appropriate to examine Figure 6 for average values and standard deviation. The flexural test fracture surfaces presented in Figure 7 show differences in the failure mechanism. Before conditioning, the surface indicates more brittle fracture and failure appears in form of matrix cracking, while conditioned samples showed failure in terms of fiber sliding (enclosed in ovals) and fiber pull-out as indicated by the deep holes (marked by arrows) in Figure 7. This is possibly because the fibers swell after the moisture uptake, creating stress concentrations on the interface leading to debonding between WF and the matrix during loading. Indications of some polymer plasticizing could also be noticed on the sliding surfaces of conditioned samples. Figure 6 demonstrates graphically the two important properties of the flexural test (stiffness and maximum stress) showing the effect of moisture uptake, while numerical values of flexural properties are presented Table 5 and Table 6 for the nanocomposites and the WPCs, respectively.

### 3.5. Differential Scanning Calorimetry

**Figure 8:**
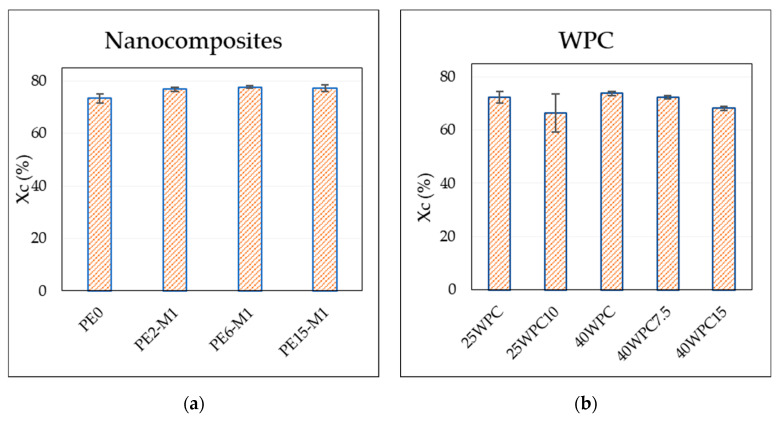

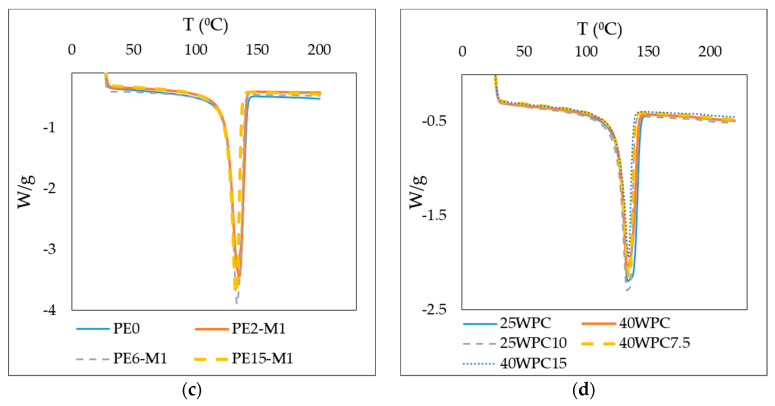
DSC results of the tested materials. It is worth noting that number of specimens tested was different for the nanocomposite than the WPC and WPC nanocomposite, which is why the range of scatter values varies. (**a**) Average degree of crystallinity of the nanocomposites; (**b**) Average degree of crystallinity of the WPCs; (**c**) The endothermic peaks from the second heating segment for the nanocomposites (heating rate of 10 °C/min); (**d**) The endothermic peaks of the second heating segment for the WPCs (heating rate of 10 °C/min).

The degree of crystallinity is shown in Figure 8 for the nanocomposites and WPC at different GNPs and wood content. From Figure 8a, no significant difference in the case of nanocomposites is found. Some difference in crystallinity with the addition of wood reinforcement is observed (Figure 8b). This indicates that crystallinity is higher with higher wood content and it is reduced with the addition of nano-reinforcement (for the same content of wood). This is also visible in the shape of the melting peaks presented in Figure 8c,d, where the peaks of the WPC are smaller but also rather wider than those of the nanocomposites (full thermograms can be found in Figures S3 and S4). The reason can be attributed to the significant increase in the viscosity of the polymer at such high content of reinforcements, which can restrict the movement of the polymer chains obstructing them from diffusion to the crystal nuclei [33]. However, the experimental scatter is rather high, and more specimens have to be tested to exclude possible influence of material inhomogeneity. Therefore, no solid conclusions concerning the influence of reinforcement on crystallinity of matrix are made at this point.

While the degree of crystallinity is not significantly changed, Table 7 shows that the addition of the GNP reinforcement is resulting in a shift of more than 4 °C in the crystallization temperature of the nanocomposites compared to neat polymer. Crystallization starts earlier in the sample (higher temperature in the cooling stage) indicating the nucleation effect of the reinforcement [34,35]. Studies have shown both positive and negative impacts on crystallization when adding nanoparticles into the polymers [36,37]. It is possible that there is a competing mechanism that results either in a change of the crystallization rate or in the heterogeneity of the crystallization. Based on the size and distribution of the nanoparticles, they can act as nucleating agents around which spherulites start to form and grow. On the other hand, if these particles are large, they can restrict the growth of the crystals and hinder the heterogeneous crystallization [38]. Figure 8 and Table 7 show these possibly competing mechanisms where expected agglomerates at 15 wt% GNP in wood composites might hinder the formation of polymer crystals. The presence of both reinforcements has a more pronounced effect on the crystallization, since the higher amount of reinforcement provides no space for the crystals to grow and, hence, the crystallinity is further decreased (at 25WPC10 and 40WPC15). Moreover, the DSC measurements are sensitive to sample homogeneity and it is difficult to secure a representative sample for the bulk of the material at 5–12 mg weight. This has been investigated for several samples of PE6-M1 in TGA (not presented here), where the residual mass after five random measurements was always less than 6% the initial mass which leads to higher crystallinity if Equation (1) is used based on the assumption of a homogeneous sample since the amount of polymer is being overestimated.

### 3.6. Thermal Transport Properties

**Figure 9:**
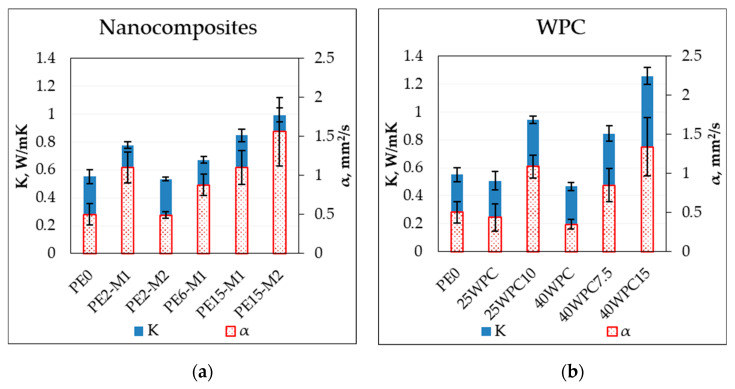
Thermal conductivity and diffusivity of (**a**) the nanocomposites; and (**b**) the wood polymer composite.

Figure 9 shows the thermal conductivity and thermal diffusivity of the samples measured by the thermal transient method. The degree of crystallinity of the polymer can be directly related to its thermal conductivity as the thermal wave can travel in the ordered structure better than the amorphous structure where the vibrational modes are localized [39]. The immediate increase in the thermal conductivity after the addition of the GNPs (>40%) is attributed to the inherent high thermal conductivity of these GNPs. Similarly, the insulative nature of the wood flour is apparent in the reduced conductivity of the wood composites with respect to the neat polymer and this reduction is larger with the increased wood content (decrease by 15% at 40 wt% wood content compared to PE0). A maximum improvement in the thermal properties of the nanocomposites is achieved at the highest GNP content for the sample PE15-M2 where the thermal conductivity and diffusivity are increased by 80% and 210%, respectively. With respect to 40WPC, the addition of 15 wt% GNP resulted in an increase of thermal conductivity and diffusivity of 168% and 243%, respectively.

There are several factors affecting the phonon transport through the nano-reinforced polymers which complicate the mechanisms of thermal conduction and their understanding. Some of these factors are the phonon scatter at the weak interfaces and the size of the conductive particles that affect the size of the contact area, chemical composition and the alignment of the polymer chains etc. [39]. Evgin et al. [34] reported the effect of aspect ratio and lateral size of the platelets on the properties of HDPE/GNPs nanocomposites. They showed that lateral size rather than aspect ratio has a larger impact on the thermal conductivity facilitated by the reduced sites for phonon scattering, and for the same lateral size, the smaller thickness results in higher conductivity due to more efficient bonding with the matrix. However, compared to the result in this study, better improvement was found for the larger aspect ratio (aspect ratio of the M2 masterbatch is ~7500 and for M1 ~2500). Many of the mentioned factors may play roles in altering the thermal conductivity of the materials in this study. For example, the gradient of crystallinity through the sample thickness as a result of the immediate exposure of the demolded surface (quenching) after compression molding compared to the gradual cooling deeper in the sample.

The synergistic effect of the two types of reinforcement is observed by the improved thermal behavior of the hybrid composite at the same amount of conductive reinforcement (40WPC15 and PE15-M1, which are made of the same GNP type). The presence of wood leads to changes in microstructure resulting in an increase of both the thermal conductivity and thermal diffusivity. Moreover, similar thermal conductivity was measured for the samples PE15-M1 and 25WPC10 despite the lower content of GNP in the latter compared to the former. This can be directly explained by the formation of conductive paths aided by the presence of wood as a scaffold for formation of conductive particle network. Once again, the samples made with the masterbatch M2 outperform that of M1, which has more phonon scattering sites dictated by the higher number of layers.

## 4. Conclusions

Modification of HDPE was achieved using HDPE-based masterbatches with high contents of graphene. Two types of GNP were used and the masterbatch with thinner platelets (fewer layer graphene sheets) showed better reinforcing performance compared to the thicker platelets. The addition of 15 wt% GNP rendered improvement in the mechanical properties to a maximum of 140% and 79% for the tensile stiffness and yield stress, respectively. A marginal effect of nanoparticles on impact strength of the wood composites was found, while improvement above 50% was seen for the nanocomposites. Composites with hybrid reinforcements (GNP and WF) showed a 243% improvement of bending stiffness.

The addition of GNPs also manifested itself by limiting the decrease of the stiffness due to the moisture. Furthermore, the mechanism of fracture of conditioned material was also altered. Some modifications observed by adding small wt% of GNPs could be achieved after certain WF loading, which indicates the possible balance between the amounts of the two reinforcements to achieve synergy with large compensation on the weight. Moreover, the modified composites showed improved thermal conductivity without compromising the mechanical properties after processing.

In general, homogeneous distribution of the nanoparticles and defects in the materials lead to consistent results from the different tests with moderate scatter. The overall improvement in the mechanical properties of the manufactured material without or with insignificant amounts of compatibilizer suggest a promising future to be utilized in more advanced applications. This can contribute to advances in relevant applications, such as thermal management of building, heated floors, or in the automotive industry utilizing more sustainable materials.

## Figures and Tables

**Table 1:** Parameters of the extrusion process.

**Extruder Zones, Temperatures and Elements**																	
**Zones**	**1**	**2**	**3**	**4**	**5**	**6**		**7**	**8**			**9**	**10**		**11**	12	
**Temp (°C)**	-	180		180	200	200		200				200	200			200	
Elements *	3 × C	2 × C + 5 × K + R + C		3 × C	2 × K + C + D	C + 2 × K + C		3 × C + D + 2 × C + K + C				K + C + K + C	6 × C			C	
**Length (mm)**	100	200		100	100	100		200				100	200			25	
**Regions**	Solid feed	Melting		Side feed	Dispersing					Homogenizing				Discharge			
**Screw Speeds and Material Flow**																	
**Material**				WPCs							Nanocomposites						
**Speed (rpm) of head screw**				round 1			round 2				120						
				300			120				-						
**Mass flow (kg/h)**				10			8				8						
**Speed (rpm) of side screw**				300							300						

* C = conveying element, K = kneading block, R = reversed conveying element, D = dispersion element.

**Table 2:** Name and composition of the studied samples.

Sample Code	HDPE (wt%)	GNP (wt%)	WF (wt%)	MAPE (wt%)
PE0	100	–	–	–
PE2-M1	98	2	–	–
PE2-M2				
PE6-M1	94	6	–	–
PE15-M1	85	15	–	–
PE15-M2				
25WPC	74	–	25	1
40WPC	58.5	–	40	1.5
25WPC10 *	63.9	9.6	25	1.5
40WPC7.5 *	50.9	7.6	40	1.5
40WPC15 *	43.5	15	40	1.5

* All WPCs reinforced with GNP reported here are produced with masterbatch M1.

**Table 3:** Moisture content of conditioned samples in terms of weight percent increase with respect to original dried, sealed samples. Results are the average measurement of three samples conditioned at 40 °C and 99% RH.

**Nanocomposite**	**PE0**	**PE2-M1**	**PE2-M2**	**PE6-M1**	**PE15-M1**	**PE15-M2**
Moisture content (%)	0.178	0.044	0.157	0.147	0.167	0.671
Time to saturation (h)	685					
**WPCs**	**25WPC**	**25WPC10**	**40WPC**	**40WPC7.5**	**40WPC15**	
Moisture content (%)	2.344	2.146	4.557	4.891	4.380	
Time to saturation (h)	1325		1693			

**Table 4:** Tensile properties of the nanocomposites and WPCs.

Sample ID	E [std]	$*\;\sigma_{max}\;[\mathrm{std}]$	$\sigma_{Yield}\;[\mathrm{std}]$	$\epsilon_{Yield}\;[\mathrm{std}]$
	(GPa)	(MPa)	(MPa)	(%)
PE0	1.89 [0.06]	22.47 [0.57]	8.4 [0.34]	0.65 [0.03]
PE2-M1	2.11 [0.11]	22.71 [0.35]	8.54 [0.15]	0.60 [0.02]
PE2-M2	2.42 [0.13]	25.61 [0.28]	9.94 [0.12]	0.61 [0.02]
PE6-M1	2.49 [0.05]	23.46 [0.32]	9.90 [0.96]	0.58 [0.01]
PE15-M1	3.85 [0.19]	26.40 [0.53]	12.84 [0.45]	0.54 [0.01]
PE15-M2	4.54 [0.35]	29.33 [1.15]	15.05 [0.93]	0.53 [0.01]
25WPC	2.88 [0.33]	26.56 [0.75]	13.60 [0.68]	0.67 [0.07]
25WPC10	4.48 [0.25]	27.66 [0.46]	16.67 [0.46]	0.57 [0.02]
40WPC	4.01 [0.23]	32.34 [1.17]	18.12 [1.50]	0.65 [0.03]
40WPC7.5	4.77 [0.27]	29.98 [1.16]	18.88 [0.85]	0.60 [0.01]
40WPC15	6.03 [0.17]	28.33 [0.94]	20.69 [0.80]	0.54 [0.01]

* For the polymer and nanocomposites, maximum stress represents the maximum values achieved during the test not the ultimate strength of the material; whereas for the WPC it is the stress at failure.

**Table 5:** Flexural properties of the nanocomposites with the moisture effect. The values 4% of strain and 25 MPa stress are selected as a common value to facilitate comparison between all samples.

Sample ID	E [std] (GPa)		$\sigma_{4\%\epsilon }\;[\mathrm{std}]\;(\mathrm{MPa})$		$\epsilon_{25\;MPa}\;[\mathrm{std}]\;(\%)$	
	* AR	* C	AR	C	AR	C
PE0	1.18 [0.03]	1.16 [0.12]	26.18 [0.81]	27.26 [1.03]	3.61 [0.25]	3.37 [0.24]
PE2-M1	1.41 [0.03]	1.43 [0.19]	28.10 [0.61]	29.67 [1.01]	3.06 [0.14]	2.81 [0.24]
PE2-M2	1.88 [0.16]	1.39 [0.09]	39.10 [1.63]	29.48 [1.66]	1.78 [0.14]	2.88 [0.33]
PE6-M1	1.57 [0.04]	1.53 [0.19]	31.36 [0.48]	32.42 [1.98]	2.48 [0.07]	2.44 [0.29]
PE15-M1	2.44 [0.09]	2.33 [0.18]	39.04 [0.39]	38.55 [1.84]	1.51 [0.01]	1.65 [0.17]
PE15-M2	2.84 [0.26]	3.00 [0.06]	43.66 [3.62]	44.12 [0.90]	1.51 [0.01]	1.2 [0.05]

* AR = as-received, C = conditioned (at T = 40 °C and RH = 99%).

**Table 6:** Flexural properties of WPC with moisture effect. The values 4% of strain and 25 MPa stress in subscript are selected as a common value to facilitate comparison between all samples.

Sample ID	E [std] (GPa)		$\sigma_{4\%\epsilon }\;[\mathrm{std}]\;(\mathrm{MPa})$		$\epsilon_{25\;MPa}\;[\mathrm{std}]\;(\%)$	
	* AR	* C	AR	C	AR	C
25WPC	1.73 [0.15]	1.74 [0.1]	38.00 [3.37]	36.37 [1.12]	1.79 [0.33]	2.14 [0.09]
25WPC10	2.78 [0.14]	3.01 [0.22]	44.83 [0.62]	47.47 [3.51]	1.14 [0.04]	1.08 [0.05]
40WPC	2.55 [0.25]	1.64 [0.07]	50.02 [2.48]	34.61 [0.33]	1.11 [0.11]	2.34 [0.05]
40WPC7.5	3.49 [0.32]	2.63 [0.08]	48.83 [3.15]	40.54 [0.39]	0.87 [0.09]	1.37 [0.03]
40WPC15	4.05 [0.32]	3.04 [0.06]	47.75 [1.48]	41.72 [0.23]	0.73 [0.02]	1.12 [0.04]

* AR = As-received, C =conditioned.

**Table 7:** DSC results of the different composites.

Sample Code	* *To* [std]	* *T*_m_ [std]	* *T*_c_ [std]	* *X*_c_ [std]
	(°C)	(°C)	(°C)	(%)
PE0	124.8 [0.45]	134.7 [1.46]	113.5 [1.00]	73.4 [1.83]
PE2	124.7 [0.27]	134.7 [0.81]	115.3 [0.54]	76.9 [0.80]
PE6	125.2 [0.39]	134.0 [0.56]	116.8 [0.47]	77.7 [0.55]
PE15	125.2 [0.45]	133.0 [0.45]	117.8 [0.73]	77.2 [1.13]
25WPC	125.6 [0.03]	133.0 [0.31]	112.0 [2.69]	72.3 [2.24]
25WPC10	126.0 [0.44]	133.3 [0.34]	115.7 [0.80]	66.4 [7.09]
40WPC	125.4 [0.05]	135.4 [1.63]	111.8 [1.30]	73.8 [0.84]
40WPC7.5	127.1 [0.04]	135.7 [0.71]	114.2 [0.73]	72.4 [0.72]
40WPC15	125.8 [0.06]	133.5 [0.48]	114.9 [0.64]	68.2 [0.87]

* *To*, *T*_m_, *T*_c_: onset, melting, and crystallization temperatures, respectively; *X*_c_: degree of crystallinity; [std]: standard deviation.

## Supplementary Materials

The following are available online at https://www.mdpi.com/1996-1944/13/9/2089/s1, Figure S1: Aspect ratio distribution of the wood flour particles used in the study obtained by analyzing images from X-Ray computer tomography, Figure S2. Schematic of the extruder showing the different zones, Figure S3. Thermograms of the pure polymer and the nanocomposites obtained from DSC test. Samples are named after the content of the graphene nanoplatelets (GNP) in them where PE refers to the polymer (HDPE), the numbers (2, 6, and 15) refers to the wt.% of the GNPs and M1 refers to the type of masterbatch (see the specifications of the masterbatch in the article), Figure S4. Thermograms of the wood polymer composites obtained by DSC. Sample nomenclator is following the style XWPCY where X = 25 or 40 refers to the content of wood and Y = 7.5, 10, 15 indicates the amount of graphene nanoplatelets (all in wt.%).
