# Supplementary material for: Characterization of Wood and Graphene Nanoplatelets (GNPs) Reinforced Polymer Composites
Source: Materials (Basel). 2020 May 1;13(9):2089. doi: 10.3390/ma13092089 (PMC7254240; doi:10.3390/ma13092089)
Supplement: Supplementary file 1 [file materials-13-02089-s001.zip › materials-784136-supplementary.docx]

Supporting Information

Characterization of Wood and Graphene Nanoplatelets (GNPs) Reinforced Polymer Composites

Zainab Al-Maqdasi ^1,^*, Guan Gong ^2^, Birgitha Nyström ^3^, Nazanin Emami ^1^ and Roberts Joffe ^1,^*

^1^ Department of Engineering Materials and Mathematics, Luleå University of Technology, SE-971 87 Luleå, Sweden; nazanin.emami@ltu.se

^2^ Rise Sicomp AB, Fibervägen 2, SE-941 26 Öjebyn, Sweden; guan.gong@ri.se

^3^ Podcomp AB, Skylvägen 1, SE-943 33 Öjebyn, Sweden; birgitha.nystrom@podcomp.se

***** Correspondence: zainab.al-maqdasi@ltu.se (Z.A.-M.); Roberts.Joffe@ltu.se (R.J.);

Tel.: +46-0-920-491055 (Z.A.-M.)

Received: 9 April 2020; Accepted: 29 April 2020; Published: date

**Figure S1.** Aspect ratio distribution of the wood flour particles used in the study obtained by analyzing images from X-Ray computer tomography.


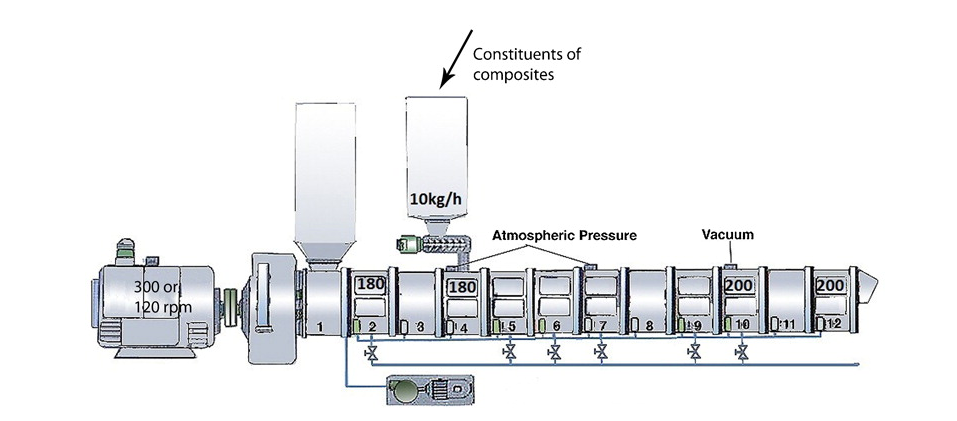


**Figure S2.** Schematic of the extruder showing the different zones [1].


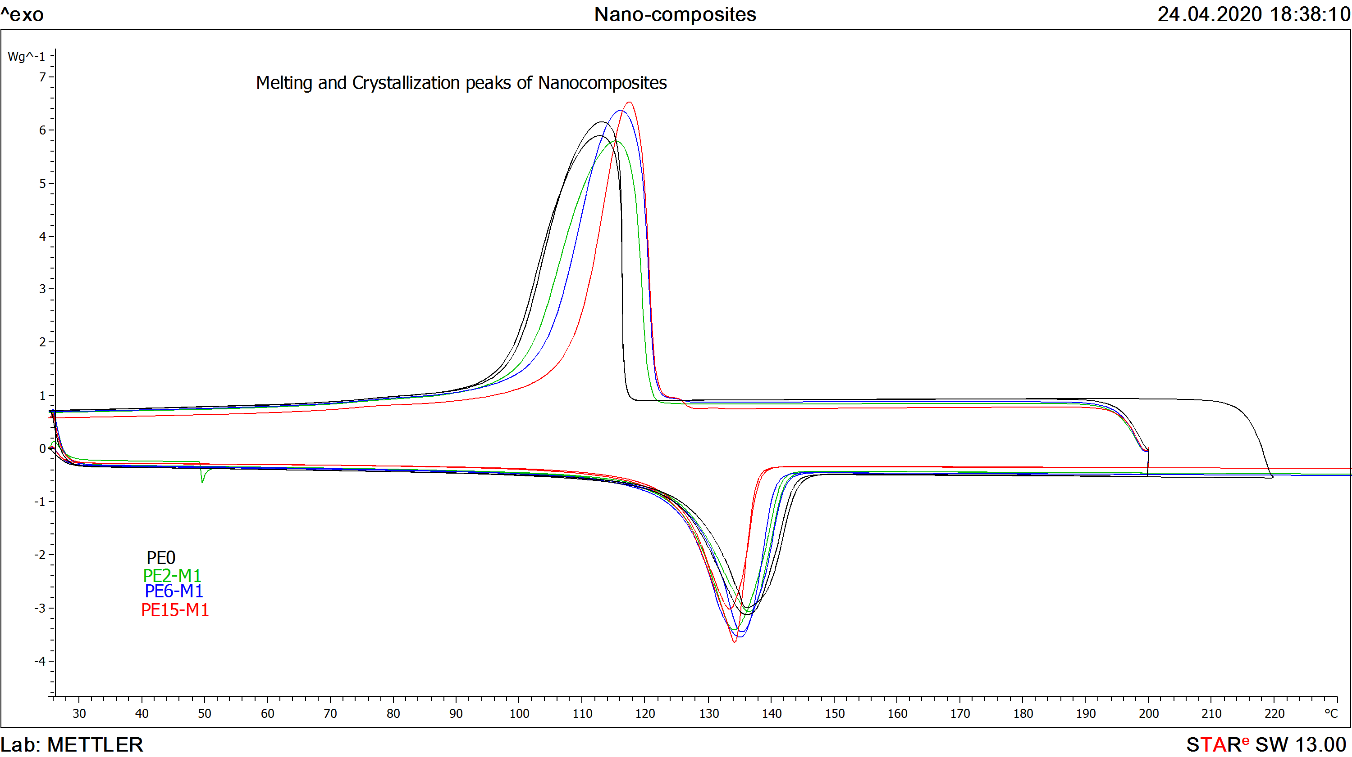


**Figure 3.** Thermograms of the pure polymer and the nanocomposites obtained from DSC test. Samples are named after the content of the graphene nanoplatelets (GNP) in them where PE refers to the polymer (HDPE), the numbers (2, 6, and 15) refers to the wt.% of the GNPs and M1 refers to the type of masterbatch (see the specifications of the masterbatch in the article).


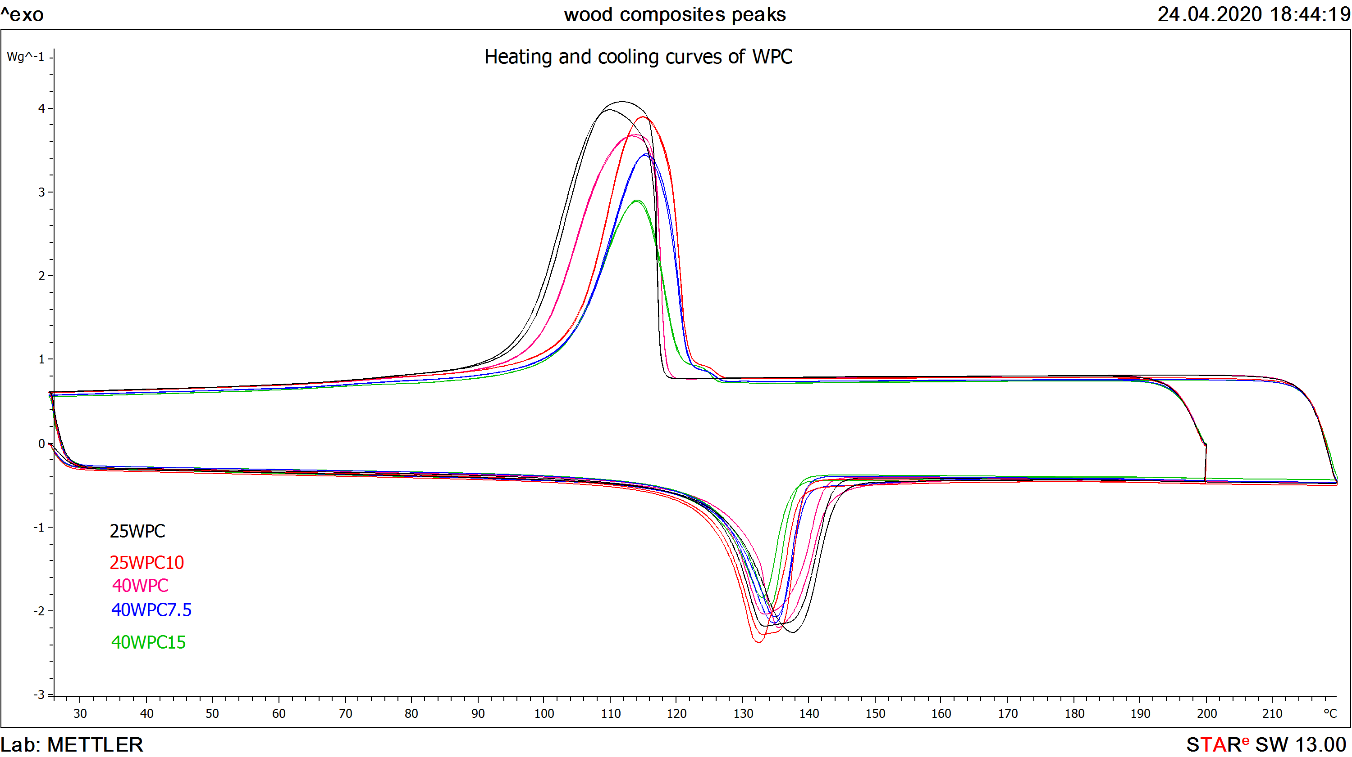


**Figure 4.** Thermograms of the wood polymer composites obtained by DSC. Sample nomenclator is following the style XWPCY where X = 25 or 40 refers to the content of wood and Y = 7.5, 10, 15 indicates the amount of graphene nanoplatelets (all in wt.%).

References

1. Gong, G.; Nyström, B.; Joffe, R. Enhanced Thermal Stability and Flame Retarding Properties of Recycled Polyethylene Based Wood Composites via Addition of Polyethylene/Nanoclay Masterbatch. *Plast. Rubber Compos.* **2013**, *42* (6), 244–255, doi: 10.1179/1743289812Y.0000000037.
